# Assessment of the changes in alveolar bone quality after fixed orthodontic therapy: A trabecular structure analysis

**DOI:** 10.15171/joddd.2016.032

**Published:** 2016-12-21

**Authors:** Abdolaziz Haghnegahdar, Hooman Zarif Najafi, Maryam Sabet, Maryam Saki

**Affiliations:** ^1^Department of Oral and Maxillofacial Radiology, School of Dentistry, Shiraz University of Medical Sciences, Shiraz, Iran; ^2^Orthodontics Research Center, Department of Orthodontics, School of Dentistry, Shiraz University of Medical Sciences, Shiraz, Iran; ^3^Student Research Committee, School of Dentistry, Shiraz University of Medical Sciences, Shiraz, Iran; ^4^Student Research Committee, Orthodontics Research Center, Department of Orthodontics, School of Dentistry, Shiraz University of Medical Sciences, Shiraz, Iran

**Keywords:** Bone, orthodontic appliances, panoramic radiography

## Abstract

***Background.*** Tooth displacement changes the periodontium. The aim of orthodontic treatment is desired tooth movement with minimum side effects on the alveolar bone quality. The aim of the present study was to assess changes of alveolar trabeculation in children, young adults and adults and the two genders.

***Methods.*** In this cross-sectional study, 63 patients who had been treated in Department of Orthodontics, School of Dentistry, Shiraz University of Medical Sciences, Shiraz, Iran, were chosen with convenient sampling method. They were divided into three groups based on their age. Their digitized panoramic radiographs (PRs) were evaluated at six interdental sites from the mesial aspect of the mandibular second molars to the distal aspect of the mandibular first premolars using a visual index. The trabeculation pattern was assigned as either dense (score 3), dense-sparse (score 2) or sparse (score 1). Data were imported to SPSS. Mean of the scores before treatment (score B) and mean of them after treatment (score A) were compared for each group with paired t-test. Changes between score B and sore A of the groups were compared using one-way ANOVA and post hoc tests.

***Results.*** Mean score A was significantly higher than mean score B in children (P = 0.001). In contrast, mean score A was significantly lower than mean score B in young adults (P = 0.003).

***Conclusion.*** Orthodontists should be cautious when treating young adults and adults regarding the probable, yet possibly temporary, negative effects of orthodontic therapy on the alveolar bone quality.

## Introduction

The term “bone quality” has been used to refer to different bone characteristics, including bone trabeculation.^1-4^ To predict the bone strength, both trabecular density and trabecular microstructure are important since high density of bone does not necessarily mean high trabecular variables such as trabecular thickness and number.^5,6^ Also the risk is higher in patients with sparse alveolar trabecular pattern.^7-9^ The structure of the trabecular bone is critical for the stability of an endosseous implant and mini-implant.^10^ Trabecular bone structure can be evaluated using different approaches. Methods of fractal dimension analysis and visual observation can be used on two-dimensional plain radiographs whereas a special imaging software is used in three-dimensional (3D) imaging modalities.^11,12^ The complexity and high cost of the 3D methods limit their application for everyday use. However, the inexpensive panoramic radiographs (PRs) and intraoral views provide information about the maxillary and mandibular bone without undue exposure.^13,14^

An increase in population knowledge and their esthetic demands leads to more orthodontic treatments among different age groups.^15^ The aim of orthodontic treatment is to move the teeth with minimum side effects on the alveolar bone quality.^16^ Tooth displacement necessarily changes the gingiva, periodontal fibers and alveolar bone.^17,18^ Orthodontic tooth movement is believed to happen either “through bone” or “with bone”. When teeth are displaced “with bone”, the amount of the alveolar bone resorption in the direction of the force balances the bone apposition, with no net loss of bone.^19^ However, hyalinization occurs and resorption begins if the pressure is too high on the periodontal ligament (PDL). Hyalinization results in the tooth movement “through bone”. Besides, the balance between apposition and resorption is disturbed, leading to a net loss of bone.^20^ Orthodontic therapy is a combination of these two types of tooth movements.^21^ Therefore, it can affect alveolar bone quality.

A few studies have been conducted to evaluate the effect of orthodontic treatment on bone quality.^18,22-24^ In the studies, density or thickness of cortical bone has been used as a measure of bone quality. Huange et al^18^ evaluated the density of one interdental area, between the left first molar and the second premolar in the maxilla and mandible, while Hsu et al^22^ assessed the density of maxillary anterior segment. In both researches, the alveolar bone density was found to decrease after orthodontic treatment. However, Patil et al^24^declared that alveolar bone density would increase after orthodontic treatment.

It is well known that patients’ gender, race and age can influence the bone metabolism.^25^ To the best of our knowledge, none of the previous studies considered the patients’ age and gender in orthodontic patients. Moreover, changes of trabecular structure after orthodontic treatment have not been evaluated previously. Therefore, this study was aimed to assess alterations in the quality of alveolar bone in mandibular posterior segment after fixed orthodontic treatment by evaluating the changes in trabecular structure in male and female subjects in different age groups.

## Methods

The research protocol of this study was approved by Ethics Committee of Shiraz University of Medical Sciences (Grant #92-01-21-6843). The patients’ data were kept confidential.

In this analytical cross-sectional study, 63 orthodontic patients, who had been referred to Department of Orthodontics, School of Dentistry, Shiraz University of Medical Sciences, were selected with convenient sampling method. They were all treated by one clinician and a similar appliance system [0.022 in, MBT prescription, Mini Master Series^TM^American Orthodontics^TM^ metal brackets (Sheboygan, WI, USA)] was used. Similar materials and strategies were used in all the subjects: type of archwire [Nickel Titanium (NiTi) (3M Unitek, Monrovia, California, USA), stainless steel (SS) (3M Unitek, Monrovia, California, USA)], elastomeric ligature (American Orthodontics, Sheboygan, WI, USA), elastomeric chains, elastics, separators and rotational wedges (G & H Orthodontics, Franklin, USA). The inclusion criteria consisted of available PRs both before and after fixed orthodontic treatment, which were diagnostically acceptable after digitization and taken at the same center and by the same machine (Plan MecaPromax, Plan Meca, Helsinki, Finland); non-extraction treatment regardless of third molars; proper oral hygiene (plaque index ≤10%); generalized moderate crowding (4‒7 mm); treatment by one clinician with the same treatment mechanics (arch expansion); similar treatment duration (1.5 years ± 6 months). Exclusion criteria consisted of patients with a history of orthognathic surgery; any systemic disease affecting bone; taking drugs with effects on bone metabolism during the treatment period; any grade of periodontal disease and alveolar bone loss before initiation of treatment; any impacted tooth; any visible anomalies and pathologic lesions of the mandible in PRs.

The patients were assigned to three age groups based on World Health Organization classification defined as below:

Group 1: children: 0‒14 years of age

Group 2: young adults: 15‒24 years of age

Group 3: adults: ≥25 years of age

The PRs before and after treatment were digitized, in grayscale mode, at 600 dpi using a flatbed scanner (Epson Expression 1600 Pro, Seiko Epson Corp., Japan). Digitization was performed to allow for image adjustments so that all the radiographs could be examined in a comparable status of contrast and light intensity. A lossless format of Tag Image File Format (TIFF) was used to save the radiographs in a storage device. The images were assessed with Image software which allows correction of both the contrast and the intensity of light (Adobe Photoshop 7.0, Adobe Systems Corporation Inc, San Jose, California, USA).

**Figure 1.**
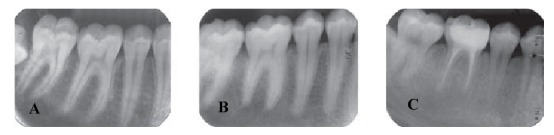
The three-scale visual analysis for assessment of bone trabeculation. A = Sparse, B = Dense-Sparse, C = Dense. Image extracted from “Pham D, Kiliaridis S. Evaluation of changes in trabecular alveolar bone during growth using conventional panoramic radiographs. Acta Odontologica Scandinavica. 2012;70:129”,^10^ reprinted by permission of Taylor & Francis Ltd, www.tandfonline.com on behalf of Acta Odontologica Scandinavica Society. v1.9 (license # 3966071191260).

The following visual assay was used to evaluate the trabeculation of the alveolar bone (Figure 1):^26^

Score 1: Sparse: Bone marrow spaces are large, especially in cervical regions.

Score 2: Dense-Sparse: In cervical regions, the trabeculation is denser and it is sparser apically.

Score 3: Dense: Bone marrow spaces are small even under the roots and the whole region has equal degree of trabeculation.

The evaluation was conducted on six interdental sites bilaterally, from the mesial side of the mandibular second molars to the distal side of the mandibular first premolars. The assessed areas were surrounded by lamina dura of the adjacent roots mesiodistally; they included the alveolar crest to 3 mm apical to the roots cervicoapically. All the PRs were mixed before and randomly to blind the examination. One experienced oral and maxillofacial radiologist carried out all the assessments of alveolar bone trabeculation.

If accurate evaluation of interdental sites was hindered by root proximity, idiopathic osteosclerosis, mandibular tori superimposition or any other anatomic structures, this interdental region was not included in the examination. Whenever trabeculation pattern was difficult to put into the defined scores, it was considered in score 2.

### Statistical analysis

Consequently, for each patient there were six numbers for trabeculation before orthodontic treatment and six numbers after the treatment. Data were imported into SPSS software (SPSS Software, Version 13.0; LEAD Technologies, Inc., Chicago, IL). Mean of the scores before treatment (score B) and after treatment (score A) were compared for each age group using paired t-test. Furthermore, changes between the mean score B and the mean sore A of the three age groups and the two genders were compared with one-way ANOVA and post hoc tests.

Since a visual scale was used to evaluate panoramic radiographs, all the images were assessed once again after 60 days by the same radiologist to examine the intra-operator errors. Kappa statistic (K-value) was used to estimate the correlation between the two sets of the reported scores and evaluate the intra-examiner reliability.

## Results

The total number of patients was 63 (42 females and 21 males). There were 33 patients consisting of 20 females and 13 males in group 1. There were twenty patients in group 2, including 16 females and 4 males. In group 3, there were 10 patients: 6 females and 4 males.

K-value for the reported scores of the two evaluations was 0.91, indicating excellent intra-examiner reliability. The scores of the first assessment were applied in the statistical analysis.

It was shown that mean score A was significantly higher than mean score B in group 1 (P = 0.001). In contrast, mean score A was significantly lower than mean score B in group 2 (P = 0.003). The difference between mean scores A and B was not statistically significant in group 3 (P = 0.587; Table 1).

**Table 1:** Mean difference in trabeculation score of alveolar bone before and after treatment in different age groups

**Age group**	**(Score A)-(Score B)**	**Number of patients**	**P-value**
**Children**	+0.18686	33	0.001
**Young adult**	-0.26668	20	0.003
**Adult**	-0.18336	10	0.587

Score A: Mean of trabeculation score after treatment; Score B: Mean of trabeculation score before treatment.

ANOVA and post hoc tests showed mean difference of trabeculation score for group 1 was significantly higher than groups 2 and 3 (P = 0.001, 0.029 respectively), but there was no statistically significant difference in mean changes of trabeculation pattern before and after treatment between groups 2 and 3 (P = 0.846; Table 2).

**Table 2:** Comparison of mean difference of trabeculation score of alveolar bone before and after treatment between different age groups

**Groups**	**Children (P-value)**	**Young adult (P-value)**	**Adult (P-value)**
**Children**	—	0.45354 (0.001)	0.37022 (0.029)
**Young adult**	−0.45354 (0.001)	—	−0.08332 (0.846)
**Adult**	−0.37022 (0.029)	0.08332 (0.846)	—

Males and females also showed no statistically significant difference in the mean changes of trabeculation pattern before and after treatment (P = 0.966).

## Discussion

The present study assessed changes in the alveolar bone quality in the mandibular posterior segment after fixed orthodontic treatment by evaluating changes in trabecular pattern in male and female subjects in different age groups.

K-value of 0.91 indicates almost perfect intra-examiner reliability.^27^ This means that the first visual evaluation of radiographs was reliable for interpretation and therefore we safely used the scores of the first assessment in statistical analyses.

Changes in the pattern of bone trabeculation and bone density are affected by several local and systemic variables.^28,29^ Attempts were made to eliminate the effect of these confounding factors by using strictly set inclusion and exclusion criteria for the study. The role of growth in bone changes during orthodontic treatment can be considered minimum during a treatment period of 1.5 years ± 6 months according to inclusion criteria. But patients’ gender, race and age can influence bone metabolism.^25^ Kiliaridis et al^10^ conducted a research to evaluate changes in alveolar bone trabeculation during growth using the same three-scale visual analysis on PRs taken two and ten years following orthodontic treatment and to discover possible differences in patterns of trabeculation in patients of various genders and ages. They found that denser trabeculation in the alveolar bone seemed to be related to age. Although they reported a slight increase in bone trabeculation after eight years in both young and adult groups, it was not statistically significant. They suggested that eight years was a short time to evaluate changes induced by the growth process. Also, in the adult group, a more significant change was found during the eight-year longitudinal assessment.

As stated earlier, the structure of trabecular bone can be evaluated by different methods: analysis of fractal dimension, visual observation and specific imaging software programs. Fractal dimension analysis and visual observation can be used on two-dimensional plain radiographs whereas a special imaging software is used in 3D imaging techniques.^11,12^ A three-scale visual analysis was applied in our study, which has been shown to evaluate trabeculation on PRs in previous studies.^10,26^ It has already been proved that PRs can be as useful as periapical radiographs in assessing trabecular pattern.^26^ Although CBCT and other advanced 3D modalities of radiography are more accurate and provide more details, taking them routinely for orthodontic purposes seems unnecessary and uncommon because of their cost and lack of availability in all the oral and maxillofacial radiology centers and also their significant radiation doses.^13,14^

Based on our results, trabecular structure of mandibular interdental areas became denser in children after fixed orthodontic treatment. In contrast, the trabeculation became sparser in young adults after treatment. Changes in trabeculation in young adults were more prominent than in children. In adults, although a slight reduction in alveolar trabeculation took place, it was not statistically significant. Therefore, considering the trabecular structure, it could be said that the alveolar bone quality of mandibular posterior segment might increase, decrease or remain unchanged after fixed orthodontic treatment in different age groups. Therefore orthodontists should be cautious when treating young adults and adults, especially in those who already have background osseous problems. Orthodontic therapy might predispose these individuals to decreased bone quality and the associated consequences such as a greater risk of fracture and instability of endosseous implants in future. As an example, a delay is suggested after completion of fixed orthodontic therapy for the placement of endosseous implants.

A few studies have been conducted to evaluate the effect of orthodontic treatments on bone quality.^18,22-24^

In most of the previous studies,^18,22,24^ bone density was used as a measure of bone quality.

Huange et al^18^ used CBCT-based degree of bone mineralization (DBM) to evaluate alterations of bone density distribution in the maxilla and mandible after orthodontic treatment in 43 patients ranging from 11.5 to 17.4 years of age. Although they did not aim at discovering whether the alveolar bone density would decrease or increase, they showed that the computed tomography (CT) attenuation parameters increased (without any statistical analysis or mentioned P-values), which means that the alveolar bone density and subsequently bone quality increased. This is consistent with our results in children. Although, no strict inclusion and exclusion criteria were set in the study of Huange et al,^18^ we considered several factors such as malocclusion type, oral hygiene, crowding, and treatment mechanics, etc, none of which being considered in sample selection in a research conducted by Huange et al.^18^

Hsu et al^22^ assessed changes of bone density around maxillary anterior teeth during orthodontic therapy on CBCT images. The alveolar bone was also divided into three regions of cervical, middle and apical and the amount of bone density changes in these three regions was compared. The density of alveolar bone reduced significantly with a mean of 24% after seven months of application of orthodontic forces but did not differ significantly between the aforementioned regions. Sample volume was eight patients, which is very small. They were only 20‒25 years of age. In this study, the mandible was evaluated and similar results about bone quality were obtained in our research for the young adults. In the study by Hsu et al,^22^ the effects of gender and age were not considered and detailed inclusion and exclusion criteria were not applied.

Patil et al^24^ evaluated alterations in density of bone using digital subtraction radiography at the crestal and subcrestal regions of interproximal alveolar bone of maxillary and mandibular posterior teeth before and after orthodontic therapy on digital PRs. The sample volume was 14 with an age range of 13‒18 years. Unlike other studies,^18,22^ alveolar bone quality improved in most regions (82.14%), with a significant increase in the density of bone. Similar results were found in our study in children. As in other studies, the investigation of Patil et al^24^ did not consider the effect of gender and age.

In our study, in growth termination group, the number of cases seemed to be insufficient (10 patients), which led to insignificant differences in statistical analysis. Furthermore, as women seek orthodontic treatment more than men, gender distribution among different groups was not even and the number of females was twice as males. This may be the reason of the absence of statistical significant between genders in this study. Therefore, future studies with larger sample sizes might show differences between the two genders. By using digitization, conventional radiographs may lose some data. Thus, it is advised to use digital imaging rather than digitization of conventional panoramic radiographs in future studies. Also, we only assessed mandibular alveolar bone. Rather than visual analysis which is an operator-dependent method, more objective approaches such as fractal dimension analysis should be used to evaluate both maxillary and mandibular alveolar trabecular pattern to enhance the accuracy in future studies.

## Conclusion

Alveolar bone quality might decrease, increase or remain unchanged after fixed orthodontic treatment in different ages. Within the limitations of the present study, it was concluded that orthodontists should be cautious when treating young adults and adults, especially those with background osseous problems, in whom orthodontic treatment might negatively affect alveolar bone quality and might at least temporarily predispose them to instability of endosseous implants or a greater risk of osseous fracture.

## Acknowledgments

The authors thank Vice-Chancellery of Shiraz University of Medical Science for supporting this research. The authors also thank Dr. Vosooghi of the Dental Research Development Center of the School of Dentistry for statistical analysis. We thank the authors of the article “Evaluation of changes in trabecular alveolar bone during growth using conventional panoramic radiographs”, Diane Pham and Stavros Kiliaridis, journal of Acta Odontologica Scandinavica, copyright © Acta Odontologica Scandinavica Society, reprinted by permission of Taylor & Francis Ltd, www.tandfonline.com on behalf of Acta Odontologica Scandinavica Society.v1.9 (license# 3966071191260). This article was derived from the thesis submitted by Maryam Sabet for the degree of Doctoral Dental Surgery.

## Authors’ contributions

The study was designed by AH and HZN, and M Sabet carried out the study procedures. The statistical analyses and explanation of data were carried out by M Sabet and M Saki. M Saki prepared the draft of manuscript. HZN and AH critically revised the manuscript. All the authors contributed to the final draft, read and approved the final manuscript.

## Funding

This work was supported and funded by Student Research Committee of Shiraz Dental School with Grant #92-6843.

## Competing interests

The authors declare no competing interests with regards to the authorship and/or publication of this article.

## Ethics approval

The research protocol of this study was approved by the Ethics Committee of Shiraz University of Medical Sciences (#92-01-21-6843).
